# Is There Still a Place for Tyrosine Kinase Inhibitors for the Treatment of Hepatocellular Carcinoma at the Time of Immunotherapies? A Focus on Lenvatinib

**DOI:** 10.3390/cancers13246310

**Published:** 2021-12-16

**Authors:** Marie Decraecker, Caroline Toulouse, Jean-Frédéric Blanc

**Affiliations:** 1 Department of Oncology, Hospital Haut Leveque-CHU Bordeaux, Avenue Magellan, 33604 Pessac, France; ctoulo200e@gmail.com (C.T.); jean-frederic.blanc@chu-bordeaux.fr (J.-F.B.); 2 INSERM U1053, BaRITOn, University Victor Segalen, 146 Rue Léo Saignat, 33000 Bordeaux, France

**Keywords:** hepatocellular carcinoma, lenvatinib, tyrosine kinase inhibitor

## Abstract

**Simple Summary:**

The combination of atezolizumab and bevacizumab has changed the therapeutic algorithm of advanced hepatocellular carcinomas. Therefore, the place of tyrosine kinase inhibitors, and among them, lenvatinib, which exhibits promising antitumour activity compared to other TKIs, needs to be redefined. Lenvatinib is still an option in case of contra-indication of immunotherapy or anti-vascular endothelial growth factor (anti-VEGF), but its place can also be discussed in second-line treatment. Otherwise, emerging strategies are currently being studied to assess the efficacy of the combination of lenvatinib with immunotherapy or loco-regional treatment for advanced HCC, as well as the efficacy of lenvatinib alone or in combination at earlier stages of the disease. The aim of this review was to define potential indications for lenvatinib treatment in different clinical situations of hepatocellular carcinoma.

**Abstract:**

The systemic treatment of hepatocellular carcinoma is changing rapidly. Three main classes of treatment are now available. Historically, multi-targeted tyrosine kinase inhibitors (TKIs) (sorafenib and lenvatinib as first-line; regorafenib and cabozantinib as second-line) were the first to show an improvement in overall survival (OS). Anti-vascular endothelial growth factor (anti-VEGF) antibodies can be used in first-line (bevacizumab) or second-line (ramucirumab) combination therapy. More recently, immuno-oncology (IO) has profoundly changed therapeutic algorithms, and the combination of atezolizumab-bevacizumab is now the first-line standard of care. Therefore, the place of TKIs needs to be redefined. The objective of this review was to define the place of TKIs in the therapeutic algorithm at the time of IO treatment in first-line therapy, with a special focus on lenvatinib that exhibits one of the higher anti-tumoral activity among TKI in HCC. We will discuss the place of lenvatinib in first line (especially if there is a contra-indication to IO) but also after failure of atezolizumab and bevacizumab. New opportunities for lenvatinib will also be presented, including the use at an earlier stage of the disease and combination with IOs.

## 1. Introduction

Hepatocellular carcinoma (HCC) is a major public health issue and, as the most common primary liver tumour, its incidence reaches one million new cases per year worldwide [1]. Although screening programs diagnose approximately 40% of HCCs at a curative stage, at least 50% of patients will be diagnosed at an intermediate or advanced stage [2]. The prognosis remains unfavourable at these later stages due to extensive tumour burden, a high frequency of liver dysfunction, and deterioration of health status, which limit access to any treatment, including systemic therapies. HCC is the second leading cause of cancer death worldwide [3,4,5].

In patients with advanced HCC (Barcelona Clinic Liver Cancer (BCLC) C) or with intermediate-stage (BCLC B) disease not eligible for, or progressing despite, locoregional therapies, systemic therapies are the gold standard of care. Preliminary results of the TARGET-HCC study demonstrated that patients with BCLC stage C were more than twice as likely to receive any systemic therapy compared to all other stages [6].

Sorafenib has been the standard treatment of care since 2007, based on improved overall survival (OS) in randomised controlled trials compared to placebo [7,8,9]. However, the management of advanced HCC has been modified since 2017 with the development of new and effective systemic treatments that improve both OS and progression free survival (PFS). Lenvatinib has been approved by the United States Food and Drug Administration and the European Medicines Agency after demonstration of the non-inferiority to sorafenib as first-line treatment for patients with advanced or unresectable HCC who have not received prior systemic therapy, based on the results of the phase III REFLECT study. Two other TKIs, regorafenib and cabozantinib, were also approved in second line after sorafenib. 

More recently, immune checkpoint inhibitors (ICIs) have shown promising results in the treatment of HCC, and the combination therapy with atezolizumab and bevacizumab is now the first-line standard of care for advanced HCCs from the IMbrave150 study, showing a clear superiority of the combination among sorafenib for OS, PFS, and quality of life.

However, TKIs (lenvatinib and sorafenib) remain an alternative in first-line standard of care in international guidelines (the European Association for the Study of the Liver, American Association for the Study of Liver Diseases, Asian Pacific Association for the Study of the Liver, European Society for Medical Oncology, and National Comprehensive Cancer Network [6,10,11,12,13]). However, TKIs are now currently indicated in patients with advanced or unresectable HCC not eligible for atezolizumab plus bevacizumab, with well-preserved liver function (Child-Pugh (CP) class A) and an Eastern Cooperative Oncology Group (ECOG) Performance Status (PS) of 0–2, [14,15]. The place of TKIs in second line after atezolizumab and bevacizumab is not consensually defined due to a lack of data from clinical trials.

This review aims to discuss the place of lenvatinib in HCC at the time of immunotherapy emergence.

## 2. Lenvatinib in the First Line Setting

### 2.1. Efficacy in Clinical Trials and in the Real Life Compared to Sorafenib

Lenvatinib is an oral inhibitor of multiple tyrosine kinase receptors, targeting vascular endothelial growth factor receptor (VEGFR1–3), fibroblast growth factor receptor (FGFR1–4), platelet-derived growth factor receptor α (PDGFR α), KIT-ligand (stem cell factor receptor), and RET (rearranged during transfection), with a distinct in vitro tyrosine kinase inhibitory profile compared to sorafenib [16,17,18,19]. 

Clinical evidence of the antitumour activity of lenvatinib was demonstrated in preclinical studies, with inhibition of both VEGF- and FGF-driven angiogenesis, and direct antiproliferative activity on liver cancer cells in vitro and in vivo, depending on the FGF-signalling pathway [20,21,22,23,24]. 

Following positive preliminary data, the REFLECT trial was conducted by Kudo et al. to compare lenvatinib with sorafenib as first-line treatment for unresectable HCC [25,26] (Table 1). 

This was a non-inferiority, multicentre, international, open-label, randomised phase 3 trial in 954 patients [14]. Patients were performance status (PS) 0–1, CP-A, and BCLC B or C without previous systemic therapy. Patients were randomised 1:1 to lenvatinib or sorafenib (478 to lenvatinib and 476 to sorafenib), stratified by region (Asia-Pacific or Western), macroscopic portal vein invasion and/or extrahepatic spread (yes or no), PS (0 or 1), and body weight (<60 or ≥60 kg). Baseline patient characteristics were similar between the two groups, except for hepatitis C virus (HCV) aetiology (lower in the lenvatinib group) and alpha-foetoprotein (AFP) baseline levels (higher in the lenvatinib group). The study was positive for all primary and secondary outcomes: the median OS was 13.6 months vs. 12.3 months (hazard ratio (HR): 0.92, 95% CI, 0.79–1.06); the PFS was 7.4 months (6.9–8.8) vs. 3.7 months (3.6–4.6) (HR: 0.66 (0.57–0.77); *p* < 0.0001); the time to progression (TTP) was 8.9 months (7.4–9.2) vs. 3.7 months (3.6–5.4) (HR: 0.63 (0.53–0.73); *p* < 0.0001); the overall response rate (ORR) according to response evaluation criteria in solid tumours (RECIST) was 24.1% (20.2–27.9) vs. 9.2% (6.6–11.8) (Odds ratio (OR) 3.13 (2.15–4.56); *p* < 0.0001), and the disease control rate (DCR) was 75.5% (71.7–79.4) vs. 60.5% (56.1–64.9). 

Subsequently, several publications confirmed significant improvements in PFS, TTP, and ORR compared to sorafenib in real life conditions reflecting a greater anti-tumoral activity [27,28,29,30,31,32].

### 2.2. Safety and Tolerability

Lenvatinib had a manageable tolerability profile in the REFLECT study. Most common treatment-emergent adverse events (TEAEs) were hypertension (42%), diarrhoea (39%), decreased appetite (34%), and decreased weight (31%). The overall safety profile was comparable to other TKIs, with similar rates of grade ≥3 TEAEs. TEAEs led to lenvatinib interruption in 40% of cases, a dose reduction in 37%, and drug withdrawal in 9% [33]. Patients treated with lenvatinib had a higher level of high blood pressure (23% vs. 14% of grades 3–4). The higher response rate and the higher frequency of severe hypertension with lenvatinib may indicate a greater anti-angiogenic effect of this drug. On the other hand, patients treated by lenvatinib exhibited a lower level of palmar-plantar erythrodysaesthesia syndrome (PPES) (3% vs. 11% of grades 3–4). Therefore, in patients with pre-existing skin diseases (outside non-healing ulcers), lenvatinib could be preferred to avoid an additional skin toxicity impacting the quality of life. Similarly, pre-ulcerative diabetic foot might benefit from the absence of the neuropathic-like pain induced by the hand–foot syndrome.

Regarding cost-effectiveness analyses, there was an increase of 0.27 life years (LY), an improvement of 0.23 quality-adjusted LY (QALY), and a decrease in costs for lenvatinib compared with sorafenib [34,35,36]. The AE treatment costs were very small and accounted for <1% of the total cost, suggesting that lenvatinib could represent a new long-awaited alternative option to sorafenib for the first-line systemic treatment of patients with unresectable HCC.

### 2.3. Predictive Biomarkers for Response to Lenvatinib

Preclinical studies demonstrated that inhibition of FGF19 signalling may play a role in the anti-tumour effects of TKIs against HCC. FGF19 is expressed in approximately one-third of HCC tissue samples and is associated with tumour aggressiveness, represented by a poorly differentiated tumour and an unfavourable prognosis [37].

In a post hoc analysis of the REFLECT study of Finn et al., lenvatinib (and not sorafenib) was associated with an increase in FGF19 and FGF23 levels at four weeks (FGF19: 55.2% vs. 18.3%, *p* = 0.0140; FGF23: 48.4% vs. 16.4%; *p* = 0.0022, respectively), suggesting efficient inhibition of the FGF signalling pathway [38]. In the lenvatinib arm, patients with a complete or partial response had a greater increase in FGF19 and FGF23 from baseline vs. non-responders (FGF19: 55.2% vs. 18.3%, *p* = 0.0140; FGF23: 48.4% vs. 16.4%; *p* = 0.0022).

Otherwise, early changes in serum FGF19 and Ang-2 (an angiogenesis regulator that plays a role through TEK tyrosine kinase and endothelium receptor levels during lenvatinib treatment) might predict clinical response and PFS. In a recent study of 74 patients (BCLC stages B and C), including patients previously treated with sorafenib or regorafenib, with a median follow-up of 157 days, significantly increased FGF19 levels and decreased Ang-2 levels were seen in lenvatinib responders compared with non-responders (ratio of FGF19 level at 4 weeks/baseline in responders vs. non-responders: 2.09 vs. 1.32, respectively, *p* = 0.0004; ratio at 8 weeks: 2.19 vs. 1.40, *p* = 0.0015) [39,40]. In multivariate analysis, the combination of serum FGF19 and Ang-2 was the most independent predictive factor for lenvatinib response (OR: 9.143; *p* = 0.0012) and PFS (HR: 0.171; *p* = 0.0240). The ability of FGF19 to predict an early lenvatinib response had a receiver operating characteristic (ROC) curve area of 0.726 at the optimal cut-off value of 1.51 for the FGF19 ratio vs. baseline, and with 68.6% specificity and sensitivity in discriminating the responder group from the non-responder group. Similarly, patients who experienced a greater decrease in Ang-2 levels were observed in the responder group compared with the non-responder group at 2 weeks (Ang-2 level ratio at 2 weeks vs. baseline: 0.709 vs. 0.893, *p* = 0.0041), 4 weeks (Ang-2 ratio: 0.584 vs. 0.810, *p* = 0.0002), and 8 weeks (Ang-2 ratio: 0.500 vs. 0.804, *p* < 0.0001).

## 3. Could Lenvatinib Compete with Atezolizumab Plus Bevacizumab?

Following the results of the recent IMbrave150 trial, the combination of atezolizumab and bevacizumab has become the first-line standard of care [41] (Table 1). 

This open-label phase 3 study compared the combination of atezolizumab/bevacizumab with sorafenib in patients with advanced unresectable and never treated HCC. The HR for mortality was 0.58 (95% CI, 0.42–0.79; *p* = 0.001) in favour of atezolizumab/bevacizumab. The PFS was 6.8 months (95% CI, 5.7–8.3) for the atezolizumab/bevacizumab group vs. 4.4 months (95% CI, 4.0–5.6) for the sorafenib group. The ORR was 27.3% vs. 11.9%, with a 5.5% complete response in the atezolizumab/bevacizumab group. Arterial hypertension was the most common Grade 3 or 4 adverse reaction in the atezolizumab/bevacizumab group (15.2% of patients).

This shift in first-line therapies led us to reconsider the place of lenvatinib in the sequential management of patients. To date, all randomised trials compare new first line therapies with sorafenib as the control arm, making it difficult to demonstrate the superiority of a specific drug.

Several recent reviews including meta-analysis aimed to compare first-line therapies of advanced HCC, most of them featuring sorafenib as the comparator [42,43,44,45]. Lenvatinib was associated with the greatest ORR benefit (OR, 3.34, 95% CI 2.17–5.14) in one study, whereas atezolizumab plus bevacizumab was superior to all other therapies including lenvatinib in others. 

A cost-utility analysis was conducted in Canada, using a partitioned survival analysis, comparing atezolizumab and bevacizumab (from a de novo network meta-analysis based on patients data and clinical input from REFLECT, extrapolated using parametric survival models), and lenvatinib and sorafenib [46]. Lenvatinib was associated with cost savings of CAD 4640 and CAD 120,095 and an improvement in quality of life of 0.15 and −0.28 vs. sorafenib, and atezolizumab and bevacizumab, respectively.

However, we still need more large cohort observational studies or randomised controlled trials to confirm the difference in efficacy and safety between lenvatinib and atezolizumab–bevacizumab combination.

Lenvatinib is first line in cases of contraindication to immunotherapy plus anti-angiogenic combinations or in special populations.

Sorafenib and lenvatinib remain two possible first-line drugs in patients with a contraindication to atezolizumab and/or bevacizumab, e.g., patients with active dysimmunity disease, cardiovascular comorbidities such as uncontrolled arterial hypertension, recent myocardial stroke, recent surgery or lack of wound healing, or marked portal hypertension (including significant oesophageal or cardio-tuberositary varices).

If atezolizumab–bevacizumab is not suitable for the patient, the choice of TKI treatment should consider clinical, radiological, and biological features: (i) tumour characteristics (number of tumours, vascular invasion, extrahepatic spread, and AFP level), (ii) underlying liver disease (CP score and portal hypertension), and (iii) general status (ECOG, comorbidities and symptoms associated with the disease). Thus, the distinct safety profile of TKI could be taken into account in the choice of TKI according to comorbidities as arterial hypertension or vascular diseases for example. 

The higher response rate and the improved TTP with lenvatinib compared to other TKI reflecting a high anti-tumoral activity could also be a selection criterion especially in symptomatic patients with a high tumour burden and threatening lesions. In patients with major liver involvement, a significant response is likely to prevent or to delay liver failure due to tumoral infiltration. Similarly, a symptomatic lesion (e.g., a painful bone metastasis) will benefit from a combination of TKIs and locoregional antalgic treatments. Less frequently in HCC, a deep response in selected patients can also lead to the consideration of curative treatments (e.g., surgical resection or ablation) through downstaging [28]. 

First-line treatment in some special situations:

### 3.1. Child-Pugh B Patients

Like most pivotal HCC phase III studies, patients with a CP-B score were not included in the REFLECT or the IMbrave150 study. 

However, the benefit of TKI in CP-B patients remains highly uncertain. Thus, results of the use of sorafenib in CP-B patients are poor according to large observational studies [47]. Moreover, in a recent multicentric prospective randomized trial reporting the role for sorafenib versus best supportive care (BSC) in CP-B patients with HCC [48], median TTP and OS were similar in the sorafenib and BSC arms; nevertheless, there was a possible benefit in the CP-B7 sub-group.

Real-world studies have attempted to describe the profile of efficacy and safety of lenvatinib within this fragile population [29,30,31,32]. CP-B patients (19%, *n* = 10 in Wang’s study, *n* = 18, 19.6% in Cheon’s study) had a similar survival compared to CP-A [29,30,31,49]. The ORR (*p* = 0.8293) and DCR (*p* = 0.7965) were not statistically different according to REFLECT inclusion criteria, for example, in Sho’s study. However, in a recent multicentre retrospective study, the OS at 12 months was significantly different between CP 5–7 (59.2%) and CP 8 patients (34.8%, *p* = 0.003) [50]. 

The data on the atezolizumab–bevacizumab combination in CP-B patients are also too scarce to rule on the ratio benefit/risk of this treatment in this weak population.

These results confirm the importance of considering hepatic function before introducing a treatment and the 2018 guidance AASLD noticed that systemic treatment could be administered in “well-selected patients with CP-B”. Systemic therapies, among them lenvatinib, could therefore represent an alternative to palliative care after discussion in the case of a multidisciplinary consultation meeting.

### 3.2. Liver Transplantation

Liver transplantation is one of the curative treatments for HCC and a classic exclusion criterion of most pivotal phase III studies. Despite the optimisation of selection criteria, post-transplant HCC recurrence remains a major cause of death, but there is no standard of care for these poor prognosis diseases. While immunotherapies are today contra-indicated after liver transplantation, TKIs appear to be the treatment of choice. Some small retrospective cohort studies have reported heterogenous effects of sorafenib on post-recurrence survival, but there are very little data concerning the use of lenvatinib in this indication [51,52,53]. A recent case report of a patient with a five-year recurrence of HCC after liver transplantation who received lenvatinib as first-line systemic therapy reported no severe AEs, no liver dysfunction, and stable blood tacrolimus levels during the entire follow-up period. 

In the pre-transplantation setting, the use of systemic treatment can be considered, particularly because of the expected long time on the waiting list. There are few data about the safety of immunotherapy in this situation. A recent review of three liver transplant recipients treated by immunotherapy before liver transplantation showed that two patients developed an acute rejection [54]. The authors also performed a retrospective cohort study with seven transplant receipts previously treated by PD-1 inhibitors of their centre (camrelizumab or pembrolizumab combined with lenvatinib). An acute rejection occurred in 14.3% of patients. Moreover, to avoid transplant rejection, immunosuppressive treatments should be prescribed at an optimal dose, and an addition of corticoids may be necessary, which could increase the risk of tumour recurrence. Overall, due to the unknown duration effects of immunotherapy, and because anti-VEGF should be stopped within six weeks before liver transplant, whereas its date is uncertain, the use of a TKI is considered in clinical practice. The prolonged time to progression with lenvatinib compared to sorafenib could be a strong argument for using lenvatinib in this situation, but this hypothesis requires further studies.

### 3.3. Severe Portal Hypertension

In case of advanced portal hypertension with an increased hemorrhagic risk, the use of anti-angiogenesis agents could be limited. For patients who cannot receive beta blockers, the protocol of ligation of esophageal varices may be long before eradication. If the risk of bleeding is increased by bevacizumab in the IMBrave study, there is no clear signal for an increase in bleeding related to portal hypertension in TKIs studies. In preclinical studies, sorafenib had beneficial effects on portocollateral circulation in cirrhotic animals [55]. Similarly, Hidaka demonstrated that sorafenib could reduce the portal venous flow in a prospective cohort study with 25 CP-A patients with advanced HCC [56]. The congestion index (portal venous area (PVA)/portal venous flow velocity (PVV)), which reflects the pathophysiological haemodynamics of portal venous system, significantly decreased (3.9 ± 1.7 vs. 3.0 ± 1.4, *p* = 0.042), but there were no significant differences in the portal venous flow velocity (PVV; cm/s). Sorafenib could therefore be used in case of severe portal hypertension. 

There are few studies regarding the safety of lenvatinib in case of advanced portal hypertension. Hidaka conducted analyses of the portal venous flow in 28 patients with advanced HCC treated with lenvatinib [57]. There was, in this study, 15% CP-B patients. The congestion index significantly worsened (0.037 ± 0.025 vs. 0.043 ± 0.024, *p* = 0.045), but there were no significant differences (*p* = 0.39) in the portal venous area (*p* = 0.665). Finally, in the REFLECT trial, as well as in a recent prospective multicenter study of 93 patients treated with lenvatinib, the efficacy and the safety of lenvatinib do not seem to be impacted by the level of portal hypertension.

Overall, there is no strong argument for choosing sorafenib rather than lenvatinib in case of severe portal hypertension, and lenvatinib could be administered in case of advanced portal hypertension (without recent bleeding).

### 3.4. Etiology of the Liver Disease

The efficacy of lenvatinib does not appear to be influenced by the etiology of the liver disease. Hiraoka et al. recently conducted a multicentre retrospective study with 103 patients with NAFLD and 427 patients with AFLD or viral -related HCC treated by lenvatinib [58] without significant difference in overall survival (OS) (20.5 vs. 16.9 months, *p*  =  0.057) between viral and NAFLD group.

On the other hand, in the IMbrave150 study, non-viral HCC etiologies (i.e., nonalcoholic fatty liver disease and alcohol) seem to be associated with a lower response rate and a lower survival with immunotherapies when compared to viral etiologies [59]. 

A recent meta-analysis of eight first-line high-quality phase three randomised clinical trials in advanced HCC (2002–2020) was published, reporting the relationship between aetiology and outcomes after systemic therapies with either TKI, anti-angiogenic, or ICI therapy [60]. Of these, five trials studied TKI/anti-VEGF (REACH, REACH-2, METIV-HCC, CELESTIAL, and JET-HCC; total of 2083 patients), and three studied immunotherapies (CheckMate-459, Journal Pre-proof 14, IMbrave150 and KEYNOTE-240; with a total of 1656 patients). The pooled HR for OS of patients with viral-related HCC treated with ICI was 0.64 (95% CI 0.5–0.83), compared with those in the standard of care group. The pooled HR for OS in patients with non-viral-related HCC treated with ICI was 0.92 (95% CI 0.77–1.11). The difference in efficacy between viral and non-viral-related HCC treated with ICI was significant (*p* of heterogeneity = 0.0259), and the effect of ICI was remarkably similar in HBV- and HCV-related HCC (HR 0.64 (95% CI 0.49–0.83) vs. HR 0.68 (95% CI 0.47–0.98), respectively). 

Since not all patients benefit from immunotherapy, the role of the aetiology of the underlying liver disease deserves to be investigated in further studies. However, even if viral-related HCCs could therefore benefit more from immunotherapy, there are currently not enough data to support the use of TKIs rather than ICI in first-line setting in non-viral-related HCCs.

## 4. Lenvatinib as Second-Line Treatment

In patients eligible for second-line therapy, after progression on atezolizumab/bevacizumab, treatment options include TKIs (sorafenib, lenvatinib, regorafenib, and cabozantinib), ramucirumab, and IO (pembrolizumab), according to local approvals. 

The development of IO as a gold standard at first line has opened new perspectives of the use of TKIs and among them lenvatinib as a second line therapy. In vitro studies of PD-1 inhibitor demonstrated that anti-PD-1 antibodies can remain bound to CD8+ T cells for more than 20 weeks [61]. The introduction of a TKI, and among them, lenvatinib, could act synergistically with anti-PD-1 antibodies even after the interruption of the immunotherapy. Aoki et al. reported encouraging results of lenvatinib when used after failure of PD-1/PD-L1 antibodies [62]. The ORR was 55.6%, the DCR was 86.1%, PFS was 10 months, and OS was 15.8 months. The OS since initiation of ICI therapy was 29.8 months, which is much longer than that conferred by lenvatinib alone as first-line therapy [63]. Yamauchi et al. conducted a study of 40 patients with HCC and reported that lenvatinib achieved a high response rate (81%) in tumours with a high expression of FGFR4 [64]. In addition, treatment with lenvatinib resulted in longer PFS in patients with a high FGFR4 expression than in those without FGFR4 expression (5.5 vs. 2.7 months, respectively), indicating that lenvatinib shows higher antitumour activity against tumours with high FGFR4 expression. However, there is a positive correlation between β-catenin mutations and FGFR4 expression, and its expression is higher in the population of tumours with WNT/β-catenin-activating mutations, which are found in approximately 20–30% of all HCCs [37,62,64,65].

Thus, even in patients who did not respond well to previous treatment with atezolizumab plus bevacizumab due to β-catenin activating mutations, subsequent treatment with lenvatinib would still provide better results due to its potent inhibitory effect on FGFR4.

A multinational, multicentre, and retrospective study reported clinical outcomes of patients who received subsequent systemic therapies after progression on atezolizumab-bevacizumab [66]. Of the 49 patients, 19 received lenvatinib. The ORR and DCR were 6.1 and 63.3%, respectively, across all patients. With a median follow-up duration of 11.0 months, PFS and OS were 3.4 months (95 CI 1.8–4.9) and 14.7 months (95% CI 8.1–21.2), respectively. Median PFS with lenvatinib was significantly longer than with sorafenib (6.1 vs. 2.5 months; *p* = 0.004), although there was no significant difference in median OS (16.6 vs. 11.2 months; *p* = 0.347). Patients treated with sorafenib had significantly more hand–foot syndromes than those treated with lenvatinib (69.0 vs. 26.3%, *p* = 0.004), while patients with lenvatinib seemed to have more fatigue and hypertension than those with sorafenib (fatigue; 42.1 vs. 17.2%, *p* = 0.058, and hypertension; 42.1 vs. 17.2%, *p* = 0.058). 

In addition, a retrospective study has recently investigated the potential use of lenvatinib (based on real-life experience and in vitro assessment) as second-line for patients intolerant to sorafenib, and as third-line for patients resistant to regorafenib [67]. The results suggest that lenvatinib is active and safe as a second/third-line treatment for unresectable HCC. Another study in a few patients treated with at least three different systemic therapies also reported the efficacy of lenvatinib as later treatment, with a tolerable toxicity profile [68].

Cabozantinib has demonstrated an improved OS and PFS in the phase 3 CELESTIAL study and is now validated for patients progressive after sorafenib [69]. Only retrospective data are available about the use of cabozantinib after ICI in HCC. In the recent multinational multicentre retrospective study of 49 patients who received subsequent systemic therapy after progression on atezolizumab-bevacizumab, only one received cabozantinib as second line [66]. There is not enough evidence in the literature to choose from the four available TKIs after failure of atezolizumab and bevacizumab.

## 5. Emerging Strategies

### 5.1. Lenvatinib and Pembrolizumab 

Lenvatinib combined with immunotherapy has demonstrated promising antitumour activity with a tolerable safety profile in preclinical and clinical studies. 

Regarding the mechanism of action of pembrolizumab and lenvatinib combination therapy, a preclinical study including in vitro and in vivo studies showed that suppression of tumour-associated macrophages, regulatory T cells, and other constituents of the tumour-suppressive microenvironment resulted in decreases in TGF-β and IL-10, the downregulation of PD-1 and Tim3, and the upregulation of ICOS and OX40, thereby inducing tumour immunity through IL-12 [70].

The combination of lenvatinib and pembrolizumab was recently approved as a second-line therapy for advanced endometrial carcinoma after the failure of systemic therapy [71]. For patients with advanced HCC, a phase 1b trial has recently shown promising results in terms of antitumoral activity, with a median OS of 22 months (95% CI, 20.4 months–not estimable), and an acceptable toxicity profile [72]. In this study, the ORR was 46.0% with mRECIST. Including a complete response in 11 patients, the median duration of response was 8.6 months, and median PFS was 9.3 months. 

A phase three study is currently underway to confirm these promising results as first-line therapy [73]. For patients with intermediate HCC, eligible for locoregional treatment, a phase three trial (LEAP-012) is investigating lenvatinib and pembrolizumab vs. placebo in combination with transarterial chemoembolisation (TACE) [74]. Recruitment for this study began in April 2020 (Supplementary Data Table S1).

### 5.2. Lenvatinib in the Intermediate Stage

TACE is one of the most widely used palliative treatments in the world. However, repeated chemoembolisation sessions can lead to impaired liver functions, limiting access to subsequent systemic treatments, and cohort studies have shown that only <20% of patients treated with chemoembolisation will be able to receive systemic treatment [75]. In addition, patients with intermediate-stage HCC constitute a very heterogeneous group, and some systems for subclassification (based in particular on CP score, beyond Milan, and up-to-seven criteria [76,77]) have been proposed to identify patients who will not benefit from TACE [78,79]. The arrival of new effective systemic treatments has led to consideration of the optimal timing for the switch from loco-regional to systemic therapies including lenvatinib as an interesting alternative to TACE as first-line treatment. 

A proof-of-concept retrospective study with a propensity matching score showed that lenvatinib could be more beneficial than TACE in HCC beyond up-to-seven criteria [80]. The lenvatinib group had a significantly higher ORR (73.3% vs. 33.3%; *p* < 0.001), a longer median PFS (16.0 vs. 3.0 months; *p* < 0.001), and a longer OS (37.9 vs. 21.3 months; HR: 0.48). Liver function, based on the albumin–bilirubin (ALBI) score, was well preserved in the lenvatinib group compared to the TACE group at the end of treatment. The preservation of liver function with lenvatinib allows full dose administration over a long period and can indirectly explain the high response rate with this treatment, optimising the access to a second-line therapy. In this same study, more than half the patients previously treated with lenvatinib were subsequently treated by TACE [81]. 

Patients who receive early lenvatinib administration may have a better prognosis than those who receive TACE [82]. A cohort study with 208 patients who were considered candidates for repeated TACE showed that cumulative survival rates were higher in patients treated with lenvatinib vs. patients treated with TACE (the 6-, 12-, 18-, and 24-month cumulative survival rates were 96.0, 90.4, 65.7, and 65.7%, respectively, vs. 94.1, 78.5, 65.3, and 48.4%, respectively, *p* < 0.001). The median survival time in the lenvatinib group was not available (95% CI, 17.1–not available), while that in the TACE group was 22.5 (95% CI, 20.9–26.7) months.

Further studies are necessary to confirm these encouraging results in patients with intermediate stage HCC, in whom TACE alone is not helpful (Supplementary Data Table S1).

### 5.3. Lenvatinib in an Adjuvant Setting

Despite a high recurrence rate after curative surgery (estimated at 70% at 5 years), there are currently no validated adjuvant therapies for patients with HCC [83,84]. The phase three, randomised, double-blind, placebo-controlled STORM trial studied sorafenib as an adjuvant treatment after surgical resection or local ablation of HCC [85]. There was no statistical difference in median recurrence-free survival between the sorafenib group vs. the placebo group (33·3 months vs. 33·7 months, respectively; *p* = 0·26), and sorafenib could not, therefore, be recommended as an adjuvant setting.

Another recent preliminary study demonstrated a potential benefit of adjuvant lenvatinib on disease-free survival and recurrence in high-risk patients with HBV-related HCC following liver transplantation [86]. A phase two study is underway to assess the efficacy and safety of adjuvant lenvatinib for patients after radical resection of HCC with a high risk of tumour recurrence [87]. In addition, the interim analysis of the LANCE study suggests that lenvatinib combined with TACE would be efficient and safe [88]. More studies attesting a benefit in recurrence-free survival of lenvatinib are necessary (Table S1).

## 6. Conclusions

**Figure 1:**
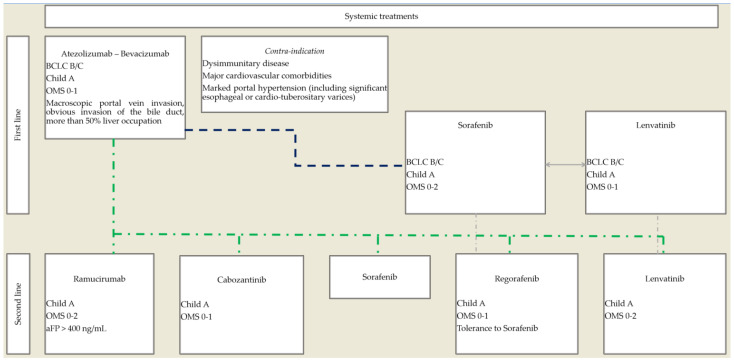
Proposed therapeutic algorithm for the use of systemic treatments in advanced, unresectable HCC.

The rapid implementation of new therapeutic options, including immunotherapies and combination therapies, has dramatically modified the treatment landscape of HCC, requiring a reassessment of sequential therapeutic strategy (Figure 1).

As the combination of bevacizumab and atezolizumab is now the first-line standard of care, the place of TKIs in monotherapy is moving to subsequent lines of treatment. Lenvatinib displays strong antitumoral activity, with the highest response rate among TKIs, and therefore, its early use in sequential therapy should be considered. Future developments could include the use of lenvatinib at an earlier stage of the disease, at stage BCLC B (in association with or vs. TACE), and first line in stage BCLC C HCC in association with immunotherapies, but further studies are required with atezolizumab and bevacizumab as a group control.

## Figures and Tables

**Table 1:** Summary of first-line validated treatments for unresectable HCC based on the results of the REFLECT and IMbrave150 trials.

	Atezolizumab-Bevacizumab	Sorafenib	Lenvatinib
Patients’ characteristics at baseline			
OMS	0/1	0/1/2	0/1
BCLC B/C, %	15%/82%	18%/82%	22%/78%
Age, %	64 (56–71)	64.9 ± 11.2	63 (20–88)
Male Sex, %	82%	87%	85%
Non-viral-related HCC aetiology, %	30%	52%	29%
Geographic region Asia vs. rest of the world, %	40%/60%	Unknown	67%/33%
Macrovascular invasion, %	38%	36%	23%
Metastatic status, %	63%	53%	61%
OS, months	NE	12.3 (10.4–13.9) //13.2 (10.4–NE)	↑ 13.6 (12.1–14.9)
ORR, %	27.3 (22.5–32.5)	9.2 (6.6–11.8)//11.9 (7.4–18.0)	24.1 (20.2–27.9)
PFS, months	6.8 (5.7–8.3)	3.7 (3.6–4.6)//4.3 (4.0–5.6)	↑ 7.4 (6.9–8.8)
TTP, months	NE	3.7 (3.6–5.4)	↑ 8.9 (7.4–9.2)
DCR, %	73.6%	55.3% to 60.5%	↑ 75.5%
TEAEs, %	98.2%	95% to 98.7%	94%
Hypertension	29.8%	24.4% to 30%	↑ 42%
Diarrhoea	18.8%	46% to 49.4%	39%
Decreased appetite	17.6%	24.4% to 27%	↑ 34%
Decreased weight	11.2%	9.6% to 22%	↑ 31%
PPES	0.9%	48.8% to 52%	27%
TEAEs grade ¾, %	56.5%	49% to 55.1%	57%
Hypertension	15.2%	14%	↑ 23%
PPES	0%	11%	3%

OS: Overall Survival; ORR: Objective Response Rate; PFS: Progression-free survival; TTP: Time To Progression; DCR: Disease Control Rate; TEAEs: Treatment-Emergent Adverse Events; PPES: Palmar-plantar erythrodysaesthesia syndrome; NE: Not Evaluated. //: REFLECT vs. IMbrave150. ↑: higher result.

## Supplementary Materials

The following are available online at https://www.mdpi.com/article/10.3390/cancers13246310/s1, Table S1. Clinical trials of lenvatinib for HCC.

## Abbreviations

AE	adverse events
AFP	alpha-foetoprotein
Anti-VEGFR	anti-vascular endothelial growth factor
BCLC	Barcelona Clinic Liver Cancer
BSC	best supportive care
CP	Child–Pugh
DCR	disease control rate
ECOG	Eastern Cooperative Oncology Group
FGFR	fibroblast growth factor receptor
HCC	hepatocellular carcinoma
HBV	hepatitis B virus
HCV	hepatitis C virus
HR	hazard ratio
ICIs	immune checkpoint inhibitors
IO	immuno-oncology
IT	immunotherapy
NE	not evaluated
OR	odds ratio
ORR	overall response rate
OS	overall survival
PFS	progression-free survival
PDGFR	platelet-derived growth factor receptor
PPES	palmar–plantar erythrodysaesthesia syndrome
PS	performance status
PVA	portal venous area
PVV	portal venous flow velocity
QALY	quality-adjusted life years
TACE	transarterial chemoembolisation
TEAEs	treatment-emergent adverse events
TTP	time to progression
TKIs	tyrosine kinase inhibitors
RECIST	response evaluation criteria in solid tumours
