# Supplementary material for: Is There Still a Place for Tyrosine Kinase Inhibitors for the Treatment of Hepatocellular Carcinoma at the Time of Immunotherapies? A Focus on Lenvatinib
Source: Cancers (Basel). 2021 Dec 16;13(24):6310. doi: 10.3390/cancers13246310 (PMC8699782; doi:10.3390/cancers13246310)
Supplement: Supplementary file 1 [file cancers-13-06310-s001.zip › cancers-1486271-supplementary.pdf]

cancers

# Is There Still a Place for Tyrosine Kinase Inhibitors for the Treatment of Hepatocellular Carcinoma at the Time of Immunotherapies? A Focus on Lenvatinib

**Marie Decraecker, Caroline Toulouse, and Jean-Frédéric Blanc**

**Table S1.** Clinical trials of lenvatinib for HCC.

| A: Phase III clinical trials. | | | | | |
|---|---|---|---|---|---|
| **Protocol** | **Identifier** | **Study Title** | **Interventions** | **Number Enrolled** | **Primary Completion** |
| Lenvatinib as Adjuvant (Stage A) | NCT04227808 | Lenvatinib as adjuvant therapy for patients who underwent radical resection of HCC with a high risk of recurrence. | Drug: lenvatinib Procedure: Surgery | 50 | Dec 2022 |
| | NCT03838796 | Lenvatinib combined with TACE for preventing recurrence in high-risk patients | Drug: lenvatinib Procedure: TACE | 482 | Dec 2021 |
| | NCT04053972 | The impact on recurrence risk of adjuvant lenvatinib for patients with HCC and microvascular invasion (MVI) after hepatectomy. | Drug: lenvatinib Procedure: Surgery | 377 | Dec 2022 |
| Lenvatinib and Locoregional treatment (Stage B or C) | NCT03838796 | Lenvatinib combined with TACE for preventing recurrence in high-risk patients | Drug: lenvatinib Procedure: TACE | 482 | Dec 2021 |
| | NCT04246177 | Lenvatinib (E7080/MK-7902) with pembrolizumab (MK-3475) in combination with TACE vs. TACE in participants with incurable/non-metastatic HCC (LEAP-012). | Drug: lenvatinib pembrolizumab Drug: oral placebo Drug: IV placebo Procedure: TACE | 950 | Apr 2025 |
| | NCT03775395 | Sorafenib combined with hepatic arterial infusion chemotherapy (HAIC) compared with lenvatinib combined with HAIC in patients with advanced HCC. | Drug: lenvatinib Drug: sorafenib Procedure: Hepatic arterial infusion chemotherapy | 250 | Dec 2021 |
| | NCT03905967 | Lenvatinib plus TACE vs. lenvatinib alone for advanced HCC patients. | Procedure: TACE Drug: lenvatinib | 336 | Apr 2023 |

| | | | | | |
|---|---|---|---|---|---|
| | NCT04687163 | Lenvatinib plus hepatic artery infusion of 130 mg/m² oxaliplatin, leucovorin, and fluorouracil vs. sorafenib or lenvatinib plus hepatic artery infusion of 85 mg/m² oxaliplatin, leucovorin, and 1200 mg/m² fluorouracil for unresectable advanced HCC: a randomised phase 3 trial. | Drug: HAIC of oxaliplatin, and 5-FU hepatic arterial infusion of oxaliplatin, leucovorin and 5-FU<br>Drug: lenvatinib or sorafenib | 400 | Dec 2022 |
| | NCT04053985 | Transarterial chemoinfusion (TAI) combined with lenvatinib in advanced HCC. | Drug: lenvatinib<br>Procedure: TAI | 206 | Dec 2022 |
| Lenvatinib and systemic treatment (Stage C) First-line | NCT04194775 | CS1003 in combination with lenvatinib compared to placebo in combination with lenvatinib as first-line therapy in subjects with advanced HCC. | Drug: CS1003+ lenvatinib<br>Drug: CS1003 placebo+ lenvatinib | 525 | Jun 2023 |
| | NCT04039607 | Nivolumab plus ipilimumab vs. standard of care (sorafenib or lenvatinib) in all randomised participants with advanced HCC who have not received prior systemic therapy. | Drug: nivolumab<br>Drug: ipilimumab<br>Drug: sorafenib<br>Drug: lenvatinib | 650 | Mar 2023 |
| | NCT04523493 | Toripalimab combined with lenvatinib vs. placebo combined with lenvatinib as 1st-line therapy for advanced HCC. | Combination drug: toripalimab combined with lenvatinib | | May 2024 |
| **B: Phase II clinical trials.** | | | | | |
| **Protocol** | **Identifier** | **Study Title** | **Interventions** | **Number Enrolled** | **Primary Completion** |
| Lenvatinib as Neo-adjuvant (Stage A) | NCT04241523 | Lenvatinib as a preoperative conversion therapy in patients with potentially resectable HCC. | Drug: lenvatinib 4 mg oral | 50 | Oct 2021 |
| | NCT04273100 | PD-1 mAb combined with lenvatinib and TACE in the treatment of BCLC B/C HCC: single arm, single centre, non- | Combination product: PD-1 mAb combined with TACE and lenvatinib | 56 | Dec 2020 |

| | | | | | |
|---|---|---|---|---|---|
| | | randomised, open label study. | | | |
| Lenvatinib + locoregional treatment (Stage B or C) | NCT04531228 | TACE-HAIC plus lenvatinib for patients with unresectable HCC. | Procedure: TACE-HAIC plus lenvatinib | 60 | Apr 2021 |
| Lenvatinib and systemic treatment (Stage C) First-line | NCT04368078 | A phase IIB clinical study. Lenvatinib plus toripalimab in patients with advanced HCC and to analyse potential biomarkers of therapeutic response. | Drug: lenvatinib Drug: toripalimab | 76 | April 2022 |
| | NCT04444167 | An open-label multi-centre phase Ib/II study. Anti-PD-1/CTLA-4 bispecific antibody AK104 plus lenvatinib as first-line therapy for patients with advanced HCC. | Biological: AK104 Drug: lenvatinib | 30 | Jan 2022 |
| | NCT03841201 | Immunotherapy with nivolumab in combination with lenvatinib for patients with multinodular, advanced stage HCC in first-line therapy. | Drug: lenvatinib Drug: nivolumab | 50 | Jul 2021 |
| | NCT04401800 | Tislelizumab in combination with lenvatinib in unresectable locally advanced or metastatic HCC. | Drug: lenvatinib Drug: tislelizumab | 66 | Sep 2022 |
| | NCT04443309 | Lenvatinib in combination with camrelizumab as first-line therapy in patients with advanced HCC. | Drug: camrelizumab Drug: lenvatinib | 53 | Aug 2022 |
| | NCT04170179 | Systemic chemotherapy of oxaliplatin, 5-fluorouracil, and leucovorin plus lenvatinib and toripalimab in | Procedure: Systemic chemotherapy Drug: lenvatinib Drug: toripalimab | 25 | Nov 2020 |

| | | | | | |
|---|---|---|---|---|---|
| | | patients with HCC with extrahepatic metastases. | | | |
| | NCT03899428 | Immune checkpoint therapy vs. target therapy in reducing serum HBsAg levels in patients with HBeAg-positive advanced HCC (VICI-5). | Drug: durvalumab<br>Drug: sorafenib<br>Drug: lenvatinib<br>Drug: regorafenib<br>Drug: cabozantinib | 30 | Dec 2021 |
| **C: Phase I clinical trials.** | | | | | |
| **Protocol** | **Identifier** | **Study Title** | **Interventions** | **Number Enrolled** | **Primary Completion** |
| Lenvatinib and systemic treatment (Stage C) | NCT04008797 | Phase 2 dose (RP2D) of E7386 in combination with lenvatinib. | Drug: E7386<br>Drug: lenvatinib | 42 | Mar 2022 |
